# New Avenues in Audio Intelligence: Towards Holistic Real-life Audio Understanding

**DOI:** 10.1177/23312165211046135

**Published:** 2021-11-09

**Authors:** Björn Schuller, Alice Baird, Alexander Gebhard, Shahin Amiriparian, Gil Keren, Maximilian Schmitt, Nicholas Cummins

**Affiliations:** 1 26522 University of Augsburg, Augsburg, Germany; 2 GLAM – Group on Language, Audio & Music, Imperial College, London, UK; 3 aud EERING GmbH, Germany; 4 Department of Biostatistics and Health Informatics, IoPPN, King’s College London, UK

**Keywords:** audio intelligence, computer audition, machine learning

## Abstract

Computer audition (i.e., intelligent audio) has made great strides in recent years; however, it is still far from achieving holistic hearing abilities, which more appropriately mimic human-like understanding. Within an audio scene, a human listener is quickly able to interpret layers of sound at a single time-point, with each layer varying in characteristics such as location, state, and trait. Currently, integrated machine listening approaches, on the other hand, will mainly recognise only single events. In this context, this contribution aims to provide key insights and approaches, which can be applied in computer audition to achieve the goal of a more holistic intelligent understanding system, as well as identifying challenges in reaching this goal. We firstly summarise the state-of-the-art in traditional signal-processing-based audio pre-processing and feature representation, as well as automated learning such as by deep neural networks. This concerns, in particular, audio interpretation, decomposition, understanding, as well as ontologisation. We then present an agent-based approach for integrating these concepts as a holistic audio understanding system. Based on this, concluding, avenues are given towards reaching the ambitious goal of ‘holistic human-parity’ machine listening abilities.

## Introduction

Typical real-world audio consists of complex combinations of overlapping events from a variety of sources, creating both clashing and harmonious relationships. Despite this complexity, humans can, with relative ease, decipher across audio (in a holistic manner) through understanding, decomposing, interpreting, and ontologisation of an abundance of potentially conveyed messages and their related semantic meanings. Historically, developments in the field of computational audio understanding (computer audition) were initially driven by speech analysis, in particular, the field of automatic speech recognition (ASR). From its inception at Bell labs in the 1950s with the ‘Audrey’ system, capable of recognising spoken digits (Davis et al., 1952), through the considerable advancements during the 1980s associated with the use of hidden Markov models (Hansen & Hasan, 2015), and to the recent deep learning revolution (Hinton et al., 2012), ASR technologies have now matured to the point where they are embedded in everyday technologies, for example, Siri^™^, Cortana^™^, and Alexa^™^. A similar transforming effect has recently occurred through deep learning, in terms of the immense increase in recognition accuracy and robustness in music analysis (e. g., Coutinho et al., 2014; Rajanna et al., 2015; Sigtia et al., 2016), and for the recognition of acoustic scenes and the detection of specific audio events (Mesaros et al., 2018).

Considering the advances in computer audition throughout the last decade (Virtanen et al., 2018), the time is now to unite these domains of audio understanding, in other words, combined disciplines of intelligent audio (e.g., interpretation, decomposition, and ontologisation) by creating a fully fledged (i.e., complete) and holistic (i.e., multi-domain) audio approach, thereby pushing this somewhat overlooked and currently underdeveloped mode of research to the forefront of intelligent machine understanding. To date, computer audition approaches have been typically mono-domain focused, with only consideration for the previously aforementioned domains of speech, music, and in general in an isolated singular manner. The view proposed here would unify these domains to truly understand and interpret audio and for the first time allow for a fine-grained level that not only recognises static traits but also the dynamic states of a given sound.

In relation to this concept of the *state* of an audio signal, initial contributions from Weninger et al. (2013), observed the acoustic similarities of more than a single audio domain, and findings showed that to a high degree there are similarities across domains, particularly between speech and music in connection with the emotional dimension of arousal. Despite this early work, there has been for some time a gap in the literature for truly holistic audio approaches, although audio decomposition approaches including *universal sound separation* (USS) from Kavalerov et al. (2019) have focused on speech and what the authors describe as universal sounds, being 2, and 3 plus additional sound to speech. From this USS approach, there is promising momentum that may be applicable to the decomposition of extremely complex audio soundscapes, towards a better understanding of those decomposed sound sources. With this in mind, in-the-wild data sources are typically unlabelled, and a holistic approach to audio is entirely needed for interpreting such data. One approach that has been applied to such data (source from YouTube) is the self-training network from Elizalde et al. (2017), which has promising results for a 10-class multi-domain problem. However, the authors highlight limitations relating to the inherent detector bias that their network developed due to the initial training data, which should be addressed when adding the many more classes of audio that are heard within a given soundscape.

The ground-breaking nature of such a holistic approach is the simultaneous understanding of the entire audible scene. Imagine, as an example, an audio scene set in a garage with two people, who are working on repairing a car while listening to music. A holistic audio analysis approach will isolate the conversation, the music, and engine noises and then assign relevant state and trait tags to each. For instance, the music genre and individual instrumentation could be recognised, the age and gender of each person and their relationship to one another determined, the car’s age, model and condition identified, and finally the repair duration logged. Such information can be obtained non-invasively, and with much lower computational costs than alternative visual modes of analysis. To this end, this information can potentially be integrated into an abundance of applications, which can then personalise aspects of security, entertainment, and household maintenance, and ultimately result in both commercial and societal benefits.

An example for both commercial and societal benefits would be to implement this holistic understanding of audio into, for example, hearing aids or ear buds. In the past, there have already been some contributions regarding hearing aids, which focused on classifying different listening environments, such as clean speech, speech in (traffic) noise, speech in babble, and music (Büchler et al., 2005; Nordqvist & Leijon, 2004). Deep learning was also recently used for this kind of task. Vivek et al. (2020) proposed a convolutional neural network (CNN)-based approach that can be utilised in hearing aids, and is able to differentiate between the five classes music, noise, speech with noise, silence, and clean speech. However, these approaches all aim for a better listening and therefore user experience. But what if more than only predefined environmental sounds would be analysed, what about the whole audio environment in a holistic manner? The possibilities which would come along with that are virtually endless. For instance, imagine a warning function in everyday traffic situations. Let us say a person using a hearing aid is in a big city with a lot of traffic and there is a car approaching. The hearing aid device could send an appropriate signal to the person. The same applies to ear buds when people are just listening to music or not paying attention to their environment in general. This might be especially useful nowadays, where the number of accidents caused by unaware pedestrians looking at their smartphones has increased (Yoshiki et al., 2017; Lu & Lo, 2017).

In the following contribution, we aim to outline the need for a more ubiquitous audio-based methodology and define the core components required to achieve the described level of holistic audio understanding. We move quickly through the state-of-the-art in the audio analysis as related to the needed aspects of such a view on the next generation of audio intelligence: audio diarisation, (audio) source separation, audio understanding, and (audio) ontologisation.

### Terminology

As our contribution is divided into a series of core concepts from the field of intelligent audio analysis, in the following we will briefly define each of these, to ensure that the reader has a precise understanding of our use of them.

- *Audio interpretation* essentially refers to the process of obtaining annotations for the audio data. This can be in a variety of methods, including categorical or dimensional labelling.
- *Audio decomposition* is the ‘break-down’ of an audio signal into its individual layers, explicitly this is the process of source separation. For example, separating overlapping speakers and removing background music.
- *Audio understanding* is the phase within an intelligent audio infrastructure in which higher-level meaning is obtained. In some cases, this may be a subjective meaning for the audio sample, which exceeds the objective truth of the sound, which was obtained during the audio interpretation stage.
- *Audio ontologisation* is the development of a knowledge base tailored specifically to audio and can be used to inform aspects of interpretation, decomposition, and understanding. For example, the sound is a bird > singing > in a woodland, in other words, source > action > environment.

Although these concepts are developed and able to function individually, we would consider that they function in a cyclical manner, with interpretation needed prior to decomposition, and these are needed for better understanding, and better understanding improving the depth of ontologisation, which we then improve interpretation.

## State-of-the-Art in Audio Analysis

### Audio Interpretation

One effort towards interpretation of audio is *acquiring annotations*. Typically, annotation is costly, time-consuming, and tedious work. In this regard, gamified intelligent crowdsourcing platforms such as iHEARu-PLAY (Hantke et al., 2016b) have been developed, to both reduce the cost associated with annotation and the mental boredom of the annotators. With help from this platform, large-scale richly labelled data collection can be performed, alleviating efficiently the scarcity of richly annotated databases. Annotation quality is assessed on small randomly selected subsets of the data by expert annotators, and by performing statistics on annotation agreements among different annotators. As a quality measure, the weighted trustability evaluator (Hantke et al., 2016a) has been introduced, which takes into consideration inter-rater agreement (much like the evaluator weighted estimator; Grimm & Kroschel, 2005), along with an individual’s ‘trustability’.

Semi-supervised active learning solutions have also been developed in the audio domain to drastically reduce human efforts by engaging models to perform annotation of the samples for which it has high confidence, while asking for human annotation if the level of confidence is low (Qian et al., 2017). Similarly, transfer learning can be applied to utilise the knowledge gained from already annotated databases and apply it to the target unannotated ones (Pan & Yang, 2009). Differences in the feature space and distribution between annotated and unannotated datasets make transferring this knowledge highly non-trivial. Transfer learning has been successfully used in applications including speech recognition of different languages (Wang et al., 2015). A range of deep neural topologies have been proposed as transfer learners, and mostly they focus on feature transfer learning. This effort can be further reduced by re-exploitation of existing data: in the domain of sound recognition, deep transfer learning has not received adequate attention.

### Audio Decomposition

*Audio decomposition* is a generalisation of speaker diarisation applied to general sound sources, for example vehicles, musical instruments, animals, or background noise types (Reynolds & Torres-Carrasquillo, 2005). This method is closely related to the task of acoustic event detection (AED) (Mesaros et al., 2018), where an audio recording is annotated with the timestamps of trained audible events, such as ‘car passing by’. The most recent advances in deep learning approaches to AED include *transfer learning* (Wang et al., 2017a), CNNs (Phan et al., 2017), *convolutional recurrent neural networks* (Amiriparian et al., 2018), and *non-negative matrix factorisation (NMF)* (Zhou & Feng, 2017).

The state-of-the-art for decomposition is mostly marked by speaker diarisation, as general audio diarisation is still gaining momentum at this time. Speaker diarisation is tagging an audio recording of several individuals with speaker turn information, that is, to provide information relating to ‘who is speaking when’. The dominating trend of the last few years in speaker diarisation research is to find suitable speaker embeddings which give a reliable multi-dimensional clustering of speech segments according to speakers. In this regard, the *i-vector* and *Gaussian mixture model-based* approaches (Anguera et al., 2012; Tranter & Reynolds, 2006) are being overtaken by deep neural network (DNN) feature representations (Bredin, 2017; Wisniewksi et al., 2017; Rouvier et al., 2015). Note that DNN-based speaker embeddings are sometimes called *d-vectors*, as opposed to *i-vectors* (Wang et al., 2017b). The advantage of DNNs for speaker diarisation is that they are capable of simultaneously learning the embeddings, that is the feature vectors describing speaker characteristics, and the scoring function, which represents the similarity between the embeddings of different segments (Garcia-Romero et al., 2017). Nevertheless, when comparing different scoring functions for i-vector embeddings, DNNs have been shown to outperform conventional scoring functions, such as *cosine similarity* and *probabilistic linear discriminant analysis* (Le Lan et al., 2017).

*Audio source separation* is the decomposition of an arbitrary audio signal into several signals with only a single audio source of interest present in each and could be a speaker, a musical instrument, a sound produced by an animal or a vehicle, or background noise, such as breaking sea waves. In most conventional approaches, a mixture-spectrogram is separated into several source spectrograms. In the past, NMF (Nikunen et al., 2018) or *non-negative tensor factorisation* (Ozerov et al., 2011) have been used for single-channel (monaural) source separation (Barker & Virtanen, 2013; Virtanen, 2007; Virtanen et al., 2011), and *independent component analysis* or *multichannel NMF* (Nikunen et al., 2018) used for multi-channel audio.

Well-studied aspects of source separation are speech denoising and speech enhancement. Previous research on speech denoising comprises *NMF* (Weninger et al., 2012), *deep NMF* (Le Roux et al., 2015), recurrent neural network (RNN)-based discriminate training (Weninger et al., 2014b), *long short-term memory-RNNs* (Weninger et al., 2015), *memory-enhanced RNNs* (Weninger et al., 2014a), and *deep recurrent autoencoders* (Weninger et al., 2014c). Latest approaches to *speech source separation* also employ different DNN types, such as *feed-forward neural networks (FFNNs)* (Naithani et al., 2016), RNNs (Huang et al., 2015; Sun et al., 2017) or *end-to-end learning* using a CNN- or RNN-autoencoder instead of the usual spectral features (Venkataramani et al., 2017). Recently, *generative adversarial nets* were found to be promising in modelling speech (Subakan & Smaragdis, 2018) and singing sources (Fan et al., 2018).

For the task of music source separation, it was found that both FFNNs and RNNs are suitable, achieving superior scores in the *signal separation evaluation campaign* music task (Uhlich et al., 2017). Latest efforts in music source separation employed *U-nets*, a CNN variant from the image processing domain (Jansson et al., 2017). Moreover, a *weakly labelled data* approach has also been proposed for the task of singing voice separation (Wang et al., 2017c). This approach utilised information about the presence or absence of singing as given by the output of a diarisation system. Notably, despite the huge amount of publications in the field of source separation, cross-domain, and thus a holistic, audio signal separation (i.e., separation of audio sources with distinct variance in character) is still largely unexplored.

### Audio Understanding

We consider audio understanding to be the task of acquiring a higher level semantic understanding of auditory scenes, sound events, speech, and music. For this task, the aim of understanding the audio goes beyond the simple identification of speech, music, objects, or events and their respective attributes. The goal, instead, should be to understand the relations between the elements of a sound scene. This understanding includes their relation to each other as well as their contextual meaning to a listener. For example, two individuals speaking loudly, followed by a door slam and then a person crying, could be understood as a heated discussion causing emotional implications. Or imagine a future possibility in which a person wearing a listening aid, ear buds, or just using their phone walking down the street and suddenly starting to breath and cough very heavily, followed by a muffled impact sound after a few seconds. This could be understood as a sudden deterioration in the person’s health state leading to a collapse. If we consider a multimodal setting, right at this moment an ambulance could be called, even before any pedestrians were able to get to the collapsed person. Or, instead of directly notifying an ambulance, the personal assistant (e.g., SIRI) could ask the person if everything is alright and wait for a response before alerting anyone. Or it would not even need to ask at all, if it recognised the voice of its ‘master’ shortly after the muffled sound.

Unlike the field of computer vision, where considerable research has been carried out on higher-levels of semantic understanding of visual tasks (e.g., visual question answering: Agrawal et al., 2017; Yang et al., 2016; image captioning: Xu et al., 2015; Lu et al., 2017), only a few works have been realised in the audio domain. One example is the recent work described in (Drossos et al., 2017), followed by their current approach in Tran et al. (2020), in which an *encoder–decoder neural network* is used to process a sequence of Mel-band energies and to compute a sequence of words that describe a given audio segment.

The already proved success of encoder-decoder sequence-to-sequence architectures for structured prediction tasks such as more general audio combined with the small number of existing works applying such models to audio understanding tasks (to the best of our knowledge) creates a window of opportunity for conducting successful research in applying encoder–decoder for the above-mentioned tasks.

### Audio Ontologisation

A core component of a holistic audio analysis, for both interpretation and understanding of audio scenes, is multi-domain audio ontologisation. A formally documented knowledge base, which provides a precise description of the concepts encompassed within a domain, with additional attributes of each concept describing possible features. Within the machine learning community, ontologisation has been widely studied and applied in the text analysis domain (Buitelaar et al., 2005), human activity recognition (Hoelzl et al., 2014), and for ‘hierarchical’ image-understanding domains (Durand et al., 2007; Deng et al., 2009; Borth et al., 2013). In the audio domain, however, due to the complexities of the everyday life soundscapes, most efforts have been focused on specific domains (Raimond et al., 2007; Han et al., 2010; Allik et al., 2016; Nakatani & Okuno, 1998).

To date, there have been scarce attempts to create complete cross-audio domain ontologisations of everyday life soundscapes. The AudioSet (Gemmeke et al., 2017) by Google has been perhaps the most interesting audio ontologisation attempt to date. It offers an ontologisation of audio events and their relationships within a sub-field, that is, classes include music, animals, and human sounds, and the corresponding dependent children are rock, dog, and whistling. AudioSet, however, does not include descriptors of the audio (e.g., the object action or emotion). This aspect aside, it does provide a platform for further and deeper ontologisation by the computer audition community. Until the release of AudioSet, the majority of works in ontologisation of audio scenes had come from studies focusing on the ontologisation of explicit audio domains, for example for music genre classification (Raimond et al., 2007), music emotion perception (Han et al., 2010), and audio features (Allik et al., 2016). Excluding AudioSet, attempts at multi-domain audio ontologisation have mainly focused on the segregation of speech and music (Nakatani & Okuno, 1998), or sound objects retrieval (Hatala et al., 2004).

To build a basis for ontologising a domain, previous research has commonly functioned in a manual nature, developing a methodology for collaborative ontology development via data mining-based visual user interfaces, such as Orange WorkFlows (OWLs) (Hilario et al., 2009). These methods create a simple ‘seed’ of basic concepts for the ontology structure (Noy et al., 2006), with further adaptations requiring huge amounts of collaborative labour, using mechanisms for carrying out discussion (e.g., polling and moderators) (Farquhar et al., 1997), something which in the long run can be time-consuming and costly. In an attempt to automate the construction of an ontology (known as ontology learning (Gotmare, 2016)), there have been efforts in the field of natural language processing, for intelligent web crawling (Maedche & Staab, 2001; Ehrig & Maedche, 2003; Ganesh et al., 2004). The web offers a mass of diverse but fragmented data sources, and targets for this can include Wikipedia, YouTube, and WordNet (Gemmeke et al., 2017). Such approaches use relevance computation (Zheng et al., 2008), to prioritise URLs of high relevance to the data which needs to be labelled, and extract metadata from social media, for example comments, tags, or titles. This textual data is then clustered into groups which may provide meaning to the associated data. To create these potential clustered groupings, unsupervised learning methods for data classification have been applied in the past (Vicient et al., 2013), as well as semi-supervised and active learning methods, in which categories are assigned based on the most informative instances (Gotmare, 2016).

Until this point, the deep ontologisation of a particular domain has been time-consuming, requiring a mass of human labour (even the state-of-the-art AudioSet ontology required a huge amount of manual human effort; Gemmeke et al., 2017). A holistic audio-domain approach will not only improve on the state-of-the-art through the inherent need for additional and more expansive audio event terminology (e.g., body acoustics, animal calls, or automotive functions), but also through more fine-grained event attributes at both the state (e.g., mood) and the trait (e.g., age) level. A starting point can be given by exploiting deep learning-based approaches for web crawling (Amiriparian et al., 2017), and clustering sourced data, as well as intelligent crowdsourcing approaches to reduce the need for manual labour, in which active learning is applied to prioritise the most informative instances (Hantke et al., 2017).

## Towards the Holistic Audio Understanding

From the above, we conclude that audio is largely being treated as a single-domain phenomenon, but the ingredients needed for a full-fledged ‘holistic’ and likewise, a more ‘human-like’ (i.e., perceiving and interpreting complex behaviours and activity in audio at speed) audio understanding are primarily available. In other words, one mainly needs to put the pieces of the puzzle together, and then feed a learning system with sufficient audio data. To overcome data sparseness, many approaches described in the literature use auditory and visual information in tandem to improve the understanding of video content. In Aytar et al. (2016), a neural network is trained on a corpus of unlabelled videos to match the representation extracted from the audio part with that extracted from the visual information by pretrained networks for object and scene classification. Facilitating such research avenues, there exist a number of video corpora that can be used for a multimodal video understanding such as Rohrbach et al. (2015) and Torabi et al. (2015).

**Figure 1.**
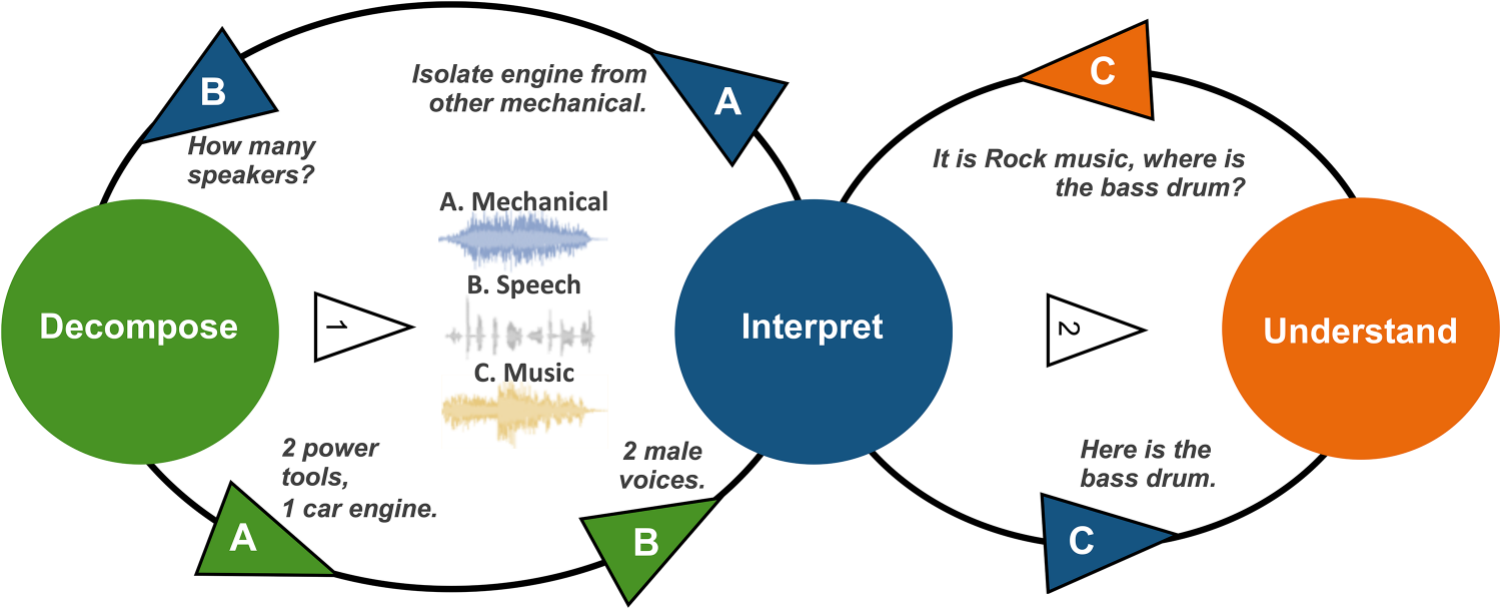
Example for an iterative approach to decompose audio interpreting on different semantic levels of ‘understanding’ to lead to an optimal ‘holistic’ audio understanding. Imagine a garage with two people working on a car and listening to music as the (audio) scene.

**Figure 2.**
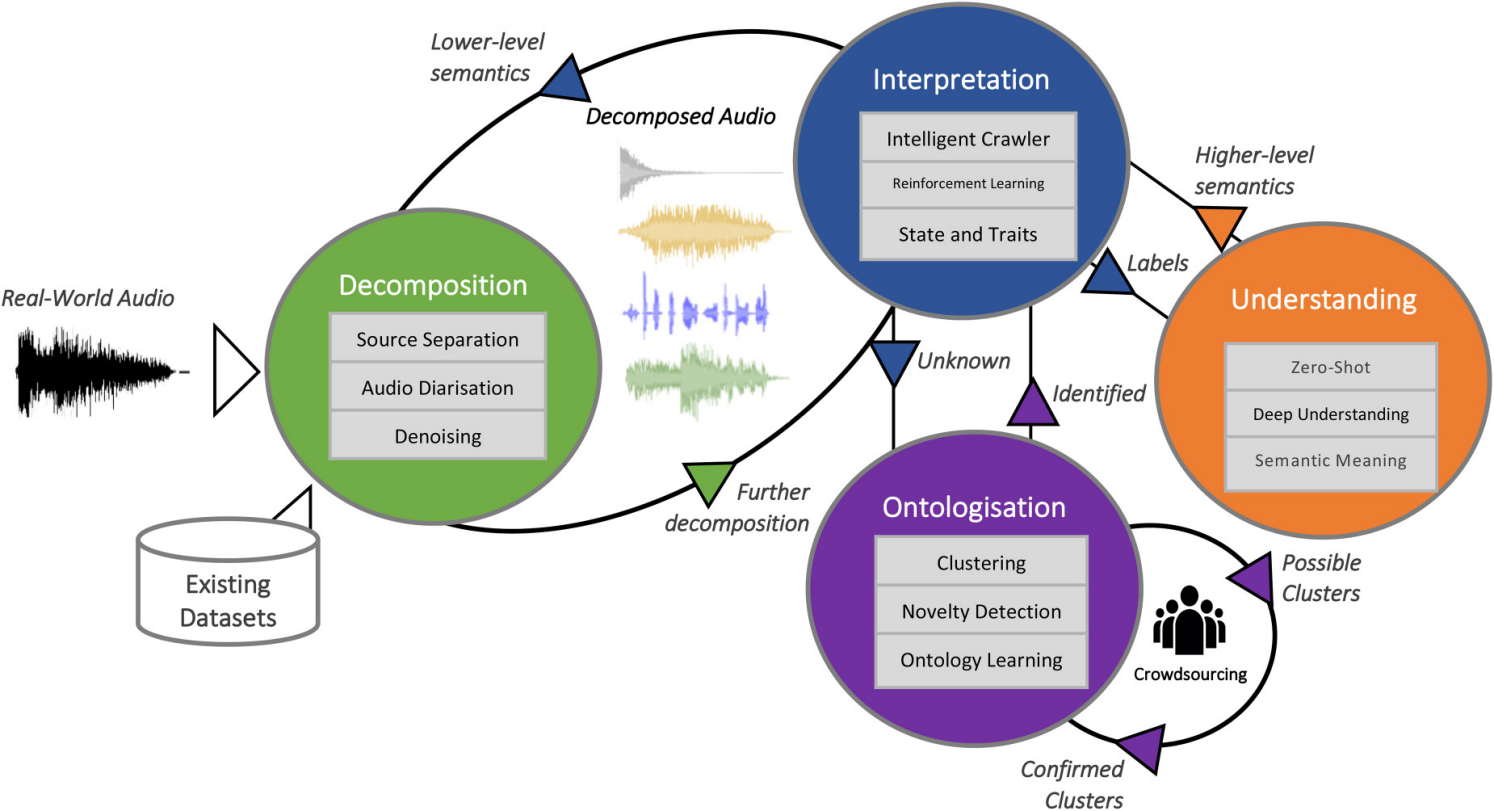
Overview of agents (decomposition, interpretation, ontologisation, understanding) and their given tasks and their interactions, as well as additional dissemination outputs.

Figure 1 visualises a potential concept towards such holistic audio intelligence. It uses an example of an audio scene, as described in the introduction. The number and type of sources present in an audio signal are not known beforehand. Hence, decomposition could be modelled as an iterative process in interaction with an interpretation component, which is providing information about the signal and indicating a request for further separation, as illustrated in Figure 1. In the proposed holistic audio-domain iterative decomposition solution, the first step would be to decompose speech, music, and sound and send separate signals to the interpretation component. The interpreter would be able to identify the types and then call the source separation again to decompose the signal events further. The source separation is aided by weak labels from the diarisation in this context, to know the temporal occurrences of the fractionally overlapping events. Finally, after the types of the audio have been classified by the interpretation component, these are analysed deeper w. r. t. states, finding that potentially parts are missing from a semantically higher perspective. This deeper analysis allows for an iterative process. Figure 2 additionally exemplifies audio ontologies that could suit the need for a complete and ‘holistic’ audio understanding. Note that the concept of state and trait assignment as known from speech analysis is consequently extended to general audio sources such as sound or music – after all, sound always has a source that has certain traits and is in certain states.

## An Agent-Based Methodology for Holistic Audio Understanding

To extend on the previously mentioned concept for holistic audio understanding, in this section we described four highly cooperative intelligent agents, which can be integrated together and developed autonomously to infer a deep comprehensive understanding of sounds.

The *interpretation agent* seeks and collects novel data by constantly exploring web sources and real-life environments (e.g., via mobile apps). The *decomposition agent* would perform fully fledged combined diarisation and source separation and associate them with a full set of basic descriptive attributes (such as loud, resonant, intermittent, continuous, noisy, etc.). An *understanding agent* will use intermediate-level attributes to recognise an unlimited number of complex sound states and traits. The *ontology agent* is then responsible for building high-level understanding of sounds (e.g., an old big car is driving on an asphalt road in a rainy weather). An overview of these interactions and cooperation is depicted in Figure 2.

### Interpretation Agent

While online multimedia archives contain an untold wealth of data, its practical application for training machine learning systems is restricted by three obstacles: (i) finding relevant recordings; (ii) segmenting into meaningful, coherent parts; and (iii) reliably labelling segments for usefulness in machine learning. To cope with these challenges, novel automated tools such as CAS2T^1^ (Amiriparian et al., 2017), have been introduced, based on a unique combination of small-world modelling of complex networks (Strogatz, 2001), unsupervised audio analysis, and crowdsourcing. Such an approach facilitates rapid training of new tasks sourced entirely via social multimedia. Concepts including curious (i.e., interpreting) multi-agent systems are promising for future research in this direction, and should be generalised and extended.

In the context of holistic audio understanding, an interpretation agent will be responsible for collecting new data samples which have high value to the other agents. The agent will do its respective tasks by crawling social media platforms and retrieving audio clips through official Application Programming Interfaces (APIs). As curiosity can be interpreted in various ways, research into defining an audio curiosity criterion is still needed, as such criterion defines what kind of audio data is novel. For these, approaches such as reconstruction-based novelty detection (e.g., autoencoder-based) (Pimentel et al., 2014) may be applied in the audio-domain, where more than one distinct type of curiosity criteria will be investigated. For example, a curiosity criterion can check if the novel sample can be considered for the development of ontology (by the ontology agent).

Different variants of a curious collection algorithm can be developed, using versatile techniques, to explore the frontiers of automatic data collection in the age of big data. Once the curiosity criterion is defined, a curious collection algorithm can go into action. The role of a curious collection algorithm is to enable fast identification of ‘related’ multimedia data from online resources. A curious collection algorithm will be a circular three-stage procedure, in which a model learns methods to better collect data. At the first stage, the agent will be exploring the different web pages of a social media platform, following a path that is determined by a parameterised path-determining-model.

As the agent travels along its path in the social media platforms, it can collect possible candidate audio samples for the next stage of the algorithm. In the second stage, these audio samples will be evaluated with respect to the curiosity criterion, to determine whether each candidate sample should be added to the database or discarded. At the third stage, the parameters of the path-determining-model from the first stage will be updated, to allow it to find audio samples that better match the curiosity criteria in the next round of actions.

Another variant of the curious interpretation agent is to use deep reinforcement learning techniques (Mnih et al., 2015) to collect the desired novel audio data from social media. In this variant, a DNN (the path-determining-model) will be conditioned on a current video’s metadata, and will decide upon the next action to take: explore a related video, apply a search operation using a new search term, etc. The metadata can include a video’s name, tags, related videos, etc. The audio samples from the videos which the agent encounters along its path will be evaluated with respect to a curiosity criterion. This evaluation can be done by feeding the audio samples into an already trained separate DNN, accepting samples on which this classifier has predictions with low confidence, and discarding the rest.

To speed up learning for the curious collection algorithm, its circular three-stage procedure will be considered for multiple interpretation agents in parallel. As was done in Mnih et al. (2016), multiple agents can explore social media platforms simultaneously, updating the parameterised path-determining-model asynchronously.

### Decomposition Agent

The task of a decomposition agent is threefold: (i) intelligent sound source separation of soundscapes with varied levels of polyphony, (ii) ontologically driven diarisation of separated concepts, and (iii) audio attribute specification for separated sounds.

Traditional sound event detection is a rapidly developing research field that deals with the complex problem of describing and understanding sounds in everyday soundscapes. State-of-the-art sound event detection systems involve locating and recognising sounds with an audio-detection onset and offset for system-known sound event instances (Mesaros et al., 2016). The complexity of state-of-the-art sound event detection systems varies with the simplest being detection of a sequence of sounds separated in the time domain. More complex systems are able to decode polyphonic sound events with multiple overlapping sounds, as is usually the case in our everyday environment (Mesaros et al., 2016). Unlike state-of-the-art sound event detection systems, the proposed decomposition agent models not only system-known sound events but also system-unknown sound concepts. Sound concepts without semantic and ontologic information will be introduced to the interpretation and ontology agent. Sound concepts include not only linguistic description of the sound event, but also can describe its states and traits.

The first task for the audio decomposition agent is an intelligent sound source separation. Instead of using state-of-the-art context-dependent sound event detection (Heittola et al., 2013), the ontology agent provides agent-prior information on the possible concepts (if available from semantic analysis of the descriptions). Corresponding prior information could significantly increase the quality of sound source separation for polyphonic soundscapes. Methods such as Bayesian neural networks can also be considered for an optimal balance of modelling prior and posterior information during the decomposition of real-life polyphonic sound concepts.

The second task for this agent is an advanced sound diarisation. Sound diarisation will be established as a new research field that can be specified from its common case, speaker diarisation, the process of separating an audio signal input into homogeneous segments according to its source identity (speaker identity in the case of speaker diarisation). The source identity of detected audio sources will be interpreted by the ontology agent.

The third task of the agent is responsible for the description of individual audio attributes (such as ‘live’, ‘nature’ or ‘mechanical’, ‘monotonic’ or ‘variable’). The audio attributes can then be defined and organised by the ontology agent and provide prior knowledge for the learning agent for zero-shot learning.

### Understanding Agent

The task of this agent is to learn and understand sophisticated and detailed categories of sound sources that act as the basic units of complex soundscapes. This novel source-centric perspective on acoustic scenes moves the analysis to a new dimension with a high capacity for describing real-life environments. The categories of sound sources could be richly related to the sound source traits and states. Usually, the traditional supervised machine learning algorithms for sound categorisation require large quantities of annotated data to be continuously collected for any novel soundscape under scrutiny. Obtaining such rich annotations with sound source traits and states is a challenging task due to the effort and expense of careful annotations. Going to real-life highly complex soundscapes, such as a street with many sound sources, annotations are becoming harder to obtain due to the number of possible traits and states. Describing real environments is the future of intelligent system operation.

**Figure 3.**
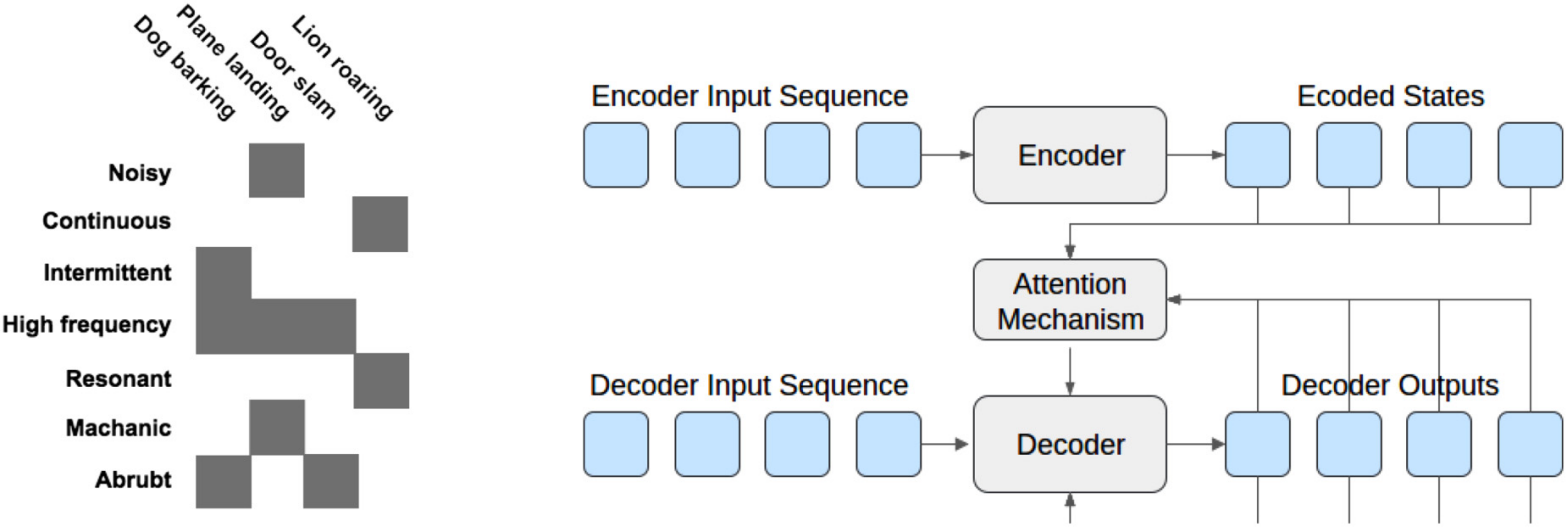
(Left) Example attribute vectors for zero-shot learning. (Right) Structure of an encoder–decoder neural network with an attention mechanism.

In the absence of rich annotations, the understanding agent can use zero-shot learning, where the combination of existing categories and semantic, cross-concept mappings between them allows for novel classifications without the need for new typical examples. Despite its maturity in the field of visual object recognition, zero-shot learning has not yet been explored for the categorisation of complex sounds. Integrating zero-shot learning enables the complex handling of unlimited numbers of sounds that continuously emerge from real environments. The understanding agent will operate on segmented and separated sounds together with their basic attributes provided by the audio decomposition agent with the goal to learn the most suitable sound traits and states. For instance, a sound described as noisy, mechanical, continuous, high-frequency, and resonant could refer to the sound of a faulty engine (left) Figure 3. In addition to zero-shot learning, deep transfer learning can be considered by the understanding agent to transfer the knowledge gained from labelled corpora to the domain of an unlabelled corpus, for example, to carry the characteristics of indoor soundscapes to outdoor ones and vice versa. This will help to process large volumes of unlabelled datasets with minimal human annotation efforts. Moreover, novel DNN techniques such as deep encoder–decoder neural networks with attention mechanisms to perform direct sequence-to-sequence mapping of sounds to their natural language descriptions, possibly in an end-to-end manner, will be investigated. Figure 3 (right) shows the block structure of this model.

The understanding agent can also utilise novel forms of the team’s dynamic active crowdsourcing (Zhang et al., 2015) to provide reliable feedback and control over the outcomes. Crowdsourcing is based on the belief that the aggregated results of work performed by numerous ‘non-experts’ approaches the quality of the same work performed by a few experts, and at a fraction of the cost. Crowdsourcing workers traditionally operate in an independent manner, however, advancing crowdsourcing by exploring techniques such as active crowdsourcing, which is inspired by the concept of ‘human swarming’ (Rosenberg et al., 2016), is worth considering. The integration of swarm intelligence into crowdsourcing would enhance the cooperation and interaction between the crowd members.

### Ontology Agent

The task of this agent is fourfold: (i) semantic analysis of user queries or on the description of novel crawled samples, (ii) detection of novel concepts from the previous task, (iii) building and expansion of a universal ontology that describes a soundscape, its state, and its relationship to other soundscapes, and (iv) providing prior information to the understanding agent (for both training and classification).

**Figure 4.**
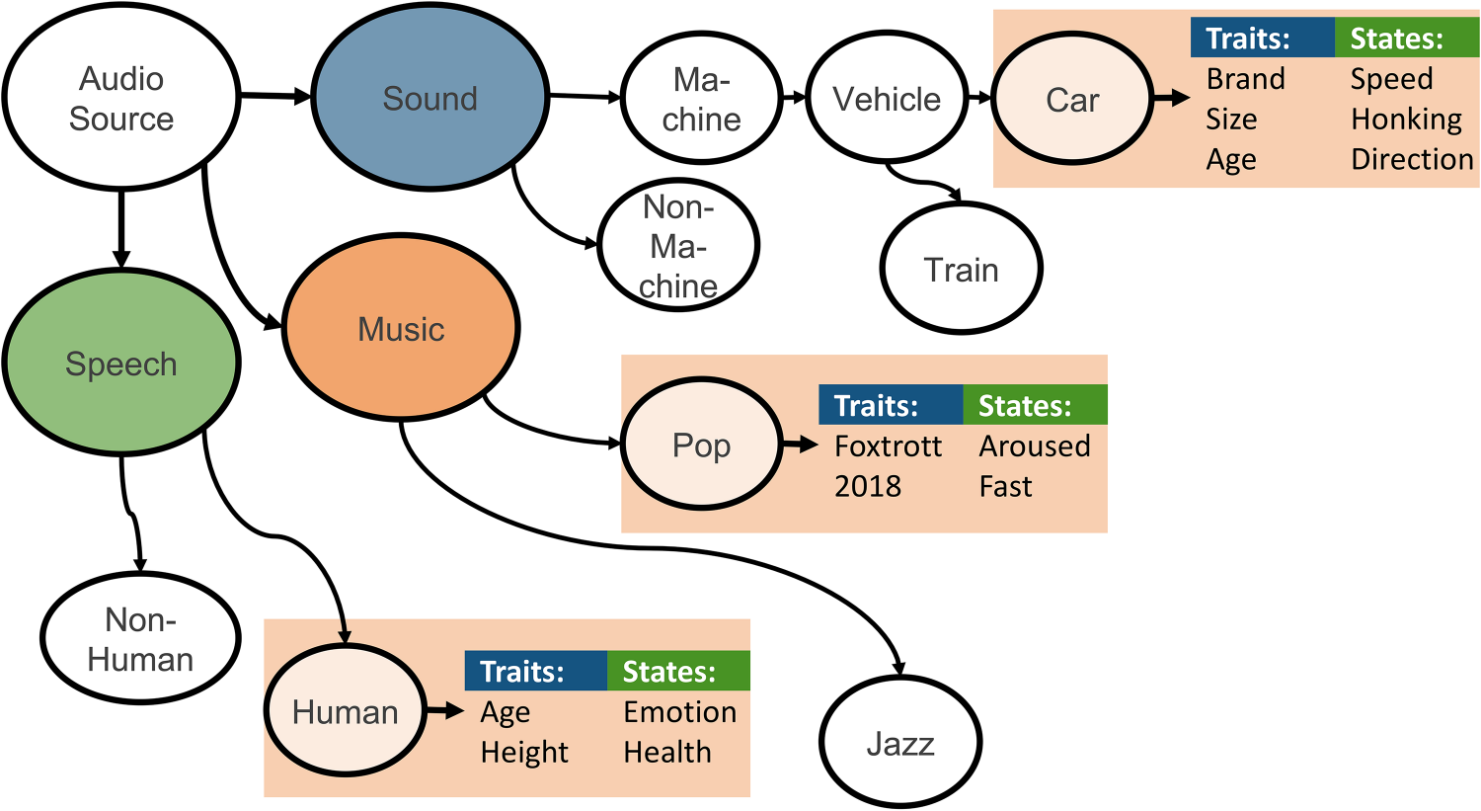
Example of an ontology that consequently attributes audio sources states and traits – not only for speech as is the current usual state-of-literature. In this depiction, we see that the audio source is decomposed into three sub-sources; speech, music, and sound, which are then each further decomposed. For example, one of the ‘sound’ sources is noted as being mechanical, vehicle, car, and the car is further labelled for its brand, as well as current action (e.g., speed).

Extending novel approaches to create an evolving ontology (such as deep ontology learning; Petrucci et al., 2016) on audio concepts (traits, states, and onomatopoeia) with less human effort, by crawling certain websites (such as Wikidata.org and dbpedia.org), interacting with the interpretation agent (novelty detection and crawling web), and the understanding agent (0-shot learning, transfer learning, and crowdsourcing). Figure 4 presents an example of ontology related to the driving scenario. Although focusing on English language audio concepts (and as standard semantic web ontology language: OWL) may provide more known-data sources, an ontological approach should be adapted for other languages. These approaches are better understood through the consideration of the following scenarios:

- Manual expansion: given that new sound databases are being generated by different communities, the content, the tags, and the labels will be used to expand the ontology and create new deep classifiers.
- Web-crawling: a web-crawler searches textual contents to find new subcategories or concepts (e.g., ‘dog is an animal’) and requests the intelligent crawler of the interpretation agent to collect audio samples related to that concept (e.g., ‘dog’). The classifier related to the super concept (e.g., ‘animal’) will be updated and tuned to incorporate the new class label.
- Query based: a user asks a query (‘I need a classifier for animals [dog, cat, bird]’). The user query analyser extracts the categories to be looked for. In case one of these categories is not yet in the ontology, the previous approach will be used to collect data and build classifiers.
- Crowdsourcing: for labelling unknown (novel) audio which is collected by the understanding agent, the ontology agent will help to narrow down possible labels (through top-down classification) to be shown to the crowd as suggestive labels. Moreover, crowdsourcing can alternatively suggest new labels and therefore, ontology can be expanded.

In relation to the entire holistic audio system, the ontology is useful for the selection of appropriate recognition models for audio, through top-down model selection, from the soundscape down to a certain trait (e.g., soundscape–mechanical–machine–vehicle–car) and state (e.g., high speed). In addition, ontology between traits and audio attributes (obtained from the decomposition agent) will empower zero-shot learning in the understanding agent. Ontology matching will be adapted and advanced (e.g., using word-embeddings; Zhang et al., 2014) to find the most similar concepts for transfer learning (in the understanding agent). There are available techniques that use ontology to enhance classification performance, such as applying deep learning approaches, from Bayesian networks (Furfaro et al., 2016) to autoencoders (Chicco et al., 2014). Moreover, integrating an ontology agent allows for further generation of use-cases. Use-cases explicitly define which categories could be of interest to classify. For example for ‘outdoor security’ as a use-case, we can define ‘human running’, ‘dog barking’, ‘angry speech’, and ‘breaking glass’ as potential threats in a ‘street’ with thousands of possible sonic combinations. Therefore, only the classifiers are applied which lead from ‘soundscape’ to these concepts.

## Conclusion

Within this article, the core contributions are three-fold (1) a detailed overview of the state-of-the-art in audio intelligence, (2) a deeper focus on audio understanding when it comes to general audio, consisting of a blend of speech and/or music and/or sound, and (3) perspectives on the next step for audio intelligence through the proposal of a fully fledged agent-based audio understanding system. From this we have surveyed each component, which we believe are crucial to lead to a general audio understanding including audio *diarisation*, source *separation*, *understanding*, and *ontologisation*. We have found from our overview of the literature that many of the approaches outlined are in a mature stage of research, and from this, we outlined a potential approach on how to combine the pieces to lead to a more advanced form of ‘holistic’ audio analysis with a rich ontology unified across the audio domains. To this end, extending our concept with detail for a fully fledged agent-based holistic audio understanding intelligence. Once realised, such an audio intelligence will find an abundance of potential applications from security to enhancing human–robot interaction, and beyond.

Next to the possibility of safety functions regarding hearing aids and ear buds, which were already introduced earlier, there are of course even more future-oriented application opportunities. Nowadays, almost everybody has their own smartphone with them wherever they are. Suppose a person’s smartphone picked up the ambient sounds from their pockets, such that a personal speech assistant, such as ALEXA or SIRI could tell them which bird is chirping or which kind of dog is currently barking at them. Additionally, the steady improvements in the field of autonomous driving are opening up even more capabilities for a holistic audio understanding. When we reach the point at which no human is needed anymore to drive a vehicle, it is the computer with whom we are communicating and which is communicating with us. Therefore, imagine a scenario in which a vehicle (e.g., a car or bus) is able to distinguish between all occurring sounds (different speakers, animals, electrical devices, etc.). It could take care of everybody in the room and even detect if a person’s health state changes. For instance, if a person suddenly starts breathing very heavily or bursts out into coughing or screams of pain, it could call an ambulance or directly drive to the next hospital while informing the hospital about its arrival and the state of the upcoming patient.

## Notes

1.
https://gitlab.com/openCoSy/CAS2T
